# Therapeutic effect and safety of Wei-Fu-Chun in the treatment of chronic atrophic gastritis: a network meta-analysis

**DOI:** 10.3389/fphar.2025.1693427

**Published:** 2025-11-20

**Authors:** Yunbo Wu, Ling Hu

**Affiliations:** Institute of Gastroenterology, Science and Technology Innovation Center, Guangzhou University of Chinese Medicine, Guangzhou, China

**Keywords:** chronic atrophic gastritis, Wei-Fu-Chun, WFC, network meta-analysis, therapeutic effect

## Abstract

**Introduction:**

Chronic atrophic gastritis (CAG) is a chronic condition characterized by a reduction in gastric mucosal glands and is often regarded as a precursor to gastric cancer. Currently, Western medicine lacks specific treatments for CAG, with management primarily focusing on symptomatic relief and, in cases involving *Helicobacter pylori* infection, eradication therapy. This study employs a network meta-analysis (NMA) to evaluate the efficacy and safety of the Chinese patent medicine Wei-Fu-Chun (WFC) in the treatment of CAG.

**Methods:**

We systematically reviewed randomized controlled trials (RCTs) comparing WFC with other interventions for CAG. The outcomes assessed included clinical effectiveness, gastrointestinal symptom scores, gastrointestinal hormone levels, and adverse reactions. The quality of the included studies was assessed with the Cochrane Handbook and GRADEpro software based on the following criteria: random sequence generation, allocation concealment, blinding of participants and personnel, blinding of outcome assessment, completeness of outcome data, and selective reporting. Risk ratios were calculated for dichotomous outcomes, and standardized mean differences with 95% confidence intervals were used for continuous variables. Funnel plots were generated to assess publication bias, and treatments were ranked using the surface under the cumulative ranking curve (SUCRA). Data analysis was performed using STATA 15.0 and Review Manager 5.3. The protocol has been registered with PROSPERO under the registration number CRD420251056533.

**Results:**

A total of 38 RCTs involving 3,844 participants were included, and 10 interventions were evaluated: conventional therapy, prokinetics, mucosal-protective agents (MPAs), acid-suppressing drugs (ASDs), *H*. *pylori* eradication therapy (HET), WFC, WFC + prokinetics, WFC + MPAs, WFC + ASDs, and WFC + HET. The majority of the included studies were evaluated as having a low risk of bias regarding randomization, attrition, reporting, and other domains, while the risk of bias remained unclear for allocation concealment and blinding. Sensitivity analysis revealed that excluding any single study had a minimal influence on the overall pooled results, and statistical heterogeneity was negligible. The NMA results indicated that WFC combined with MPA ranked the highest in overall clinical efficacy, while WFC combined with prokinetics was the most effective in restoring gastrin (GAS)/motilin (MTL) levels and alleviating symptom burden. WFC monotherapy also outperformed several Western medications (e.g., MPA, acid inhibitors, and conventional therapy) across multiple outcomes. Combinations of WFC with acid suppressants or prokinetics were associated with fewer adverse events than monotherapies, suggesting a potential reduction in drug-related side effects.

**Conclusion:**

WFC, particularly in combination with Western medications, enhances clinical efficacy and reduces the incidence of adverse events in patients with CAG. These findings support its potential as a therapeutic option for improving clinical outcomes in CAG.

**Systematic Review Registration:**

https://www.crd.york.ac.uk/prospero/, identifier CRD420251056533.

## Introduction

Chronic atrophic gastritis (CAG) is a precancerous condition characterized by chronic inflammation and progressive loss of gastric glandular cells. It represents a key stage in the Correa cascade, in which gastric carcinogenesis develops through a sequential progression from chronic gastritis to atrophy, intestinal metaplasia, dysplasia, and eventually carcinoma if untreated (Rugge et al., 2002; Sugano et al., 2015). In China, CAG affects approximately 17%–18% of the population, posing a considerable public health burden (Du et al., 2014). The major risk factors include *Helicobacter pylori* (*H. pylori*) infection, aging, smoking, high-salt diets, and autoimmune processes (Song et al., 2017; Correa et al., 2010; Park et al., 2010; Notsu et al., 2019). Clinically, CAG is often asymptomatic or presents with vague dyspeptic symptoms, and the diagnosis depends mainly on endoscopic and histopathological evaluations (Annibale et al., 2020; Pittman et al., 2015).

Current management primarily targets the underlying cause—such as *H. pylori* eradication—and symptomatic relief. However, while eradication therapy reduces inflammation and cancer risk, its ability to reverse established atrophy or metaplasia remains limited (Annibale et al., 2007; Annibale et al., 2001). Other pharmacologic options, including proton pump inhibitors (PPIs), prokinetics, and mucosal-protective agents, offer only partial symptom control and do not restore glandular structure or function (Shah et al., 2021). This therapeutic gap highlights the need for effective interventions that not only relieve symptoms but also promote histological recovery and prevent malignant transformation.

In recent years, traditional Chinese medicine (TCM) has gained attention as a complementary approach for CAG due to its multi-target, holistic therapeutic properties and favorable safety profile (Dai et al., 2017). Among these, Wei-Fu-Chun (WFC)—a patented Chinese medicine first approved in 1998, containing *Panax ginseng* C.A.Mey., *Isodon amethystoides* (Benth.) H. Hara, and *Citrus aurantium* L.—has been widely used in China for the treatment of CAG and precancerous gastric lesions (Gu et al., 2020). Experimental and clinical evidence suggest that WFC may improve gastric mucosal atrophy, modulate inflammatory responses, and inhibit *H. pylori* infection (Bian et al., 2021). A recent meta-analysis reported that WFC achieved superior histopathological improvement and symptom relief compared to standard therapies (Wang B. et al., 2023), and it is now recommended in Chinese clinical practice guidelines for CAG management (Li et al., 2023).

Despite accumulating data, most available evidence has been derived from traditional pairwise meta-analyses, which cannot simultaneously compare multiple interventions or determine their relative ranking in terms of efficacy and safety. Given the diversity of therapeutic strategies—including Western pharmacotherapies, WFC monotherapy, and various combination regimens—direct head-to-head evidence is limited. Network meta-analysis (NMA) provides a methodological advantage by integrating both direct and indirect evidence across all treatment options within a single analytical framework, allowing for a comprehensive evaluation and ranking of interventions.

Therefore, this study aims to systematically compare and rank the efficacy and safety of WFC and its combination therapies versus conventional Western treatments for CAG using a network meta-analysis of randomized controlled trials (RCTs).

## Methods

This study was conducted in accordance with the Cochrane criteria, the Preferred Reporting Items for Systematic Reviews and Meta-Analyses (PRISMA) statement (Page et al., 2021), and relevant meta-analysis guidance. The protocol has been registered with PROSPERO under the registration number CRD420251056533.

### Search strategy and study selection

A comprehensive literature search was conducted in the following databases: PubMed, EMBASE, Springer, the Cochrane Controlled Register of Trials (CENTRAL), Web of Science, and China National Knowledge Infrastructure (CNKI) from inception to 6 June 2025. No restrictions were applied regarding the language or publication date. The search terms combined controlled vocabulary and free-text words related to “chronic atrophic gastritis,” “Wei-Fu-Chun,” “WFC,” and “randomized controlled trial.” The complete search strategies for each database are provided in Supplementary File 1. Chinese-language publications were screened and translated by two bilingual investigators. To avoid duplication between Chinese and English reports of the same trial, publication details (e.g., author, year, sample size, and intervention) were cross-checked.

RCTs meeting the PICO (population, intervention, comparison, and outcome) framework (Eriksen and Frandsen, 2018) were eligible for inclusion. (1) Participants: patients with histologically confirmed CAG. (2) Interventions: pharmacological treatments including WFC or WFC combined with Western medicines. (3) Comparisons: any oral Western medicines, including prokinetics, acid-suppressing drugs (ASDs), mucosal-protective agents (MPAs), and *H*. *pylori* eradication therapy (HET). Other adjuvant therapies, such as vitamin B-12, vitacoenzyme, or placebo, were also included and classified as conventional therapies (CT). (4) Outcomes: clinical efficacy, gastrointestinal symptom scores (e.g., abdominal pain or distention, bloating, and acid reflux), gastrointestinal hormones (such as gastrin [GAS] and motilin [MTL]), serum inflammation biomarkers (such as interleukin and tumor necrosis factor [TNF]), quality of life, and adverse effects. Studies were excluded if they were abstracts (since these typically do not include complete data of the methods and results, making it difficult to extract data and assess bias), had incomplete or imprecise data, had ambiguous treatment protocols, or were not available in full text. Cross-sectional studies, reviews, and RCTs with a Jadad score of <3 were also excluded.

### Data extraction

Two authors independently extracted data from the included studies. Discrepancies were resolved by discussion, with adjudication by a third author if necessary. Extracted information included the study design, patient demographics, interventions, comparators, intervention duration, follow-up, handling of missing data, funding sources, and potential conflicts of interest. When data were missing or unclear, the original authors were contacted for clarification. If standard deviations were unavailable, they were estimated from standard errors, confidence intervals, or interquartile ranges according to the Cochrane recommendations.

### Quality evaluation

Risk of bias for RCTs was independently assessed by two authors using the Cochrane risk-of-bias tool (Savovic et al., 2014). The overall certainty of evidence was independently assessed by two authors for each stratified outcome using the GRADE methodology (Guyatt et al., 2008). Discrepancies were resolved by consultation with a third author. Risk-of-bias domains included random sequence generation, allocation concealment, blinding of participants and personnel, blinding of outcome assessment, completeness of outcome data, and selective reporting. Each domain was rated as “low risk,” “unclear risk,” or “high risk.” Heterogeneity was initially assessed qualitatively by examining the differences in study populations (e.g., age and sex), settings, interventions, durations, and outcome definitions. For studies judged to be qualitatively homogeneous, statistical heterogeneity was assessed using the chi-squared test, with *p* < 0.10 indicating significant heterogeneity. To examine the impact of study quality, sensitivity analyses were performed by excluding studies that had a high risk of bias rating.

### Data synthesis and statistical analysis

A Bayesian network meta-analysis was performed to combine direct and indirect comparisons. Random-effects models were fitted with vague (non-informative) priors. Four Markov chains were run for 50,000 iterations, with the first 10,000 discarded as burn-in. Convergence was assessed using the Gelman–Rubin diagnostic (potential scale reduction factor <1.05). Dichotomous outcomes were summarized as risk ratios (RRs) with 95% confidence intervals (CIs), and continuous outcomes were summarized as mean differences (MDs) with 95% CI. A random-effects model was applied to account for between-study variability. Network consistency was evaluated using the node-splitting method and the design-by-treatment interaction model. Publication bias and small-study effects were explored using comparison-adjusted funnel plots. Surface under the cumulative ranking curve (SUCRA) scores were calculated to rank the treatments. Statistical analyses were conducted using STATA 15.0 and Review Manager 5.3.

## Results

**FIGURE 1:**
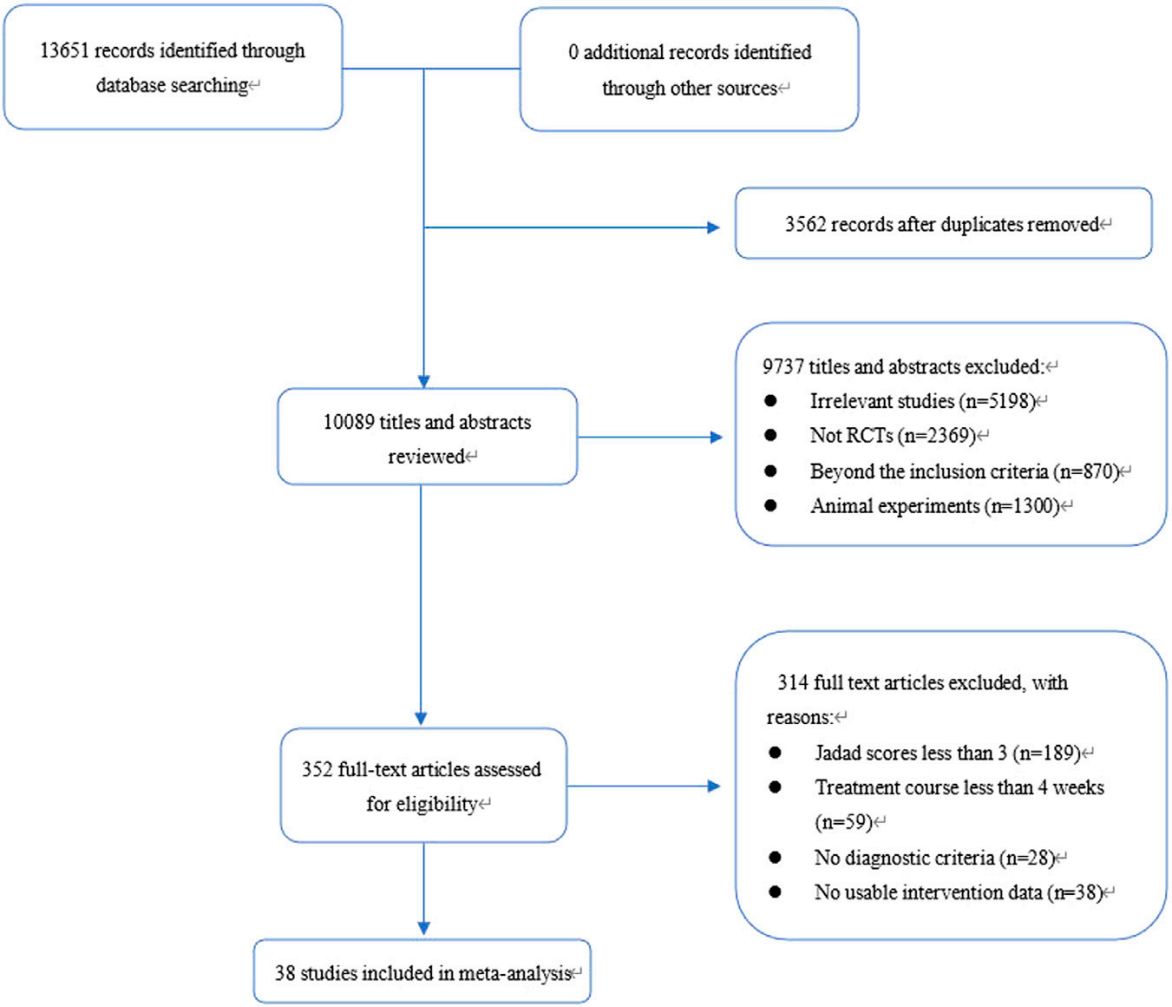
Flow diagram.

A total of 13,651 records were identified through database and manual searches. After removing duplicates and screening titles and abstracts, 352 articles were retrieved for full-text review. Of these, 38 studies met the inclusion criteria and were included in the network meta-analysis (Figure 1) (Zhang et al., 2025; Ye et al., 2024; Wang et al., 2024; Wang, 2024; Liu, 2022; Wang and Lv, 2022; Zhang, 2022; Gao et al., 2022; Bian et al., 2021; Du et al., 2021; Liang et al., 2021; Hu, 2021; Wang, 2021; Wang, 2020; Wu, 2020; Fan, 2020; Li, 2020; Yao et al., 2020; Zhu and Wei, 2019; Zhang et al., 2019; Song, 2019; Long, 2019; Xiao et al., 2018; Li and Zhao, 2018; Liu and Zhang, 2018; Xu et al., 2018; Lu et al., 2018; Fan and Qiao, 2017; He et al., 2017; Liu et al., 2017; Zhou et al., 2017; Yuan et al., 2016; Zhang et al., 2012; Nie and Sun, 2011; Fang and Du, 2011; Lin, 2011; Lin et al., 2010; Cao and Yang, 2008). The included studies involved 10 intervention types: conventional treatment (CT), prokinetics, mucosal-protective agents (MPAs), acid-suppressing drugs (ASDs), *Helicobacter pylori* eradication therapy (HET), Wei-Fu-Chun (WFC), and four combination regimens (WFC + prokinetics, WFC + MPAs, WFC + ASDs, and WFC + HET). Table 1 summarizes the basic characteristics of the selected studies, including the sample size, mean age, treatment durations, and clinical outcomes.

**TABLE 1:** Basic characteristics.

Study (year)	Age (EG/CG)	Gender (M/F)	Intervention	Comparison	Duration (weeks)	Outcome
Zhang et al. (2025)	N/A	43/37	WFC + rebamipide	WFC	12	Clinical efficacy; AE; GSS; IL-1β; SIL-2R; GAS; MTL
Wang et al. (2024)	40.06 ± 7.18/40.04 ± 7.26	41/39	WFC + PPI	PPI	8	IL-6; IL-8; TNF-α; CRP; GAS; MTL; GSS; AE
Wang et al. (2024)	39.78 ± 5.34/40.15 ± 6.17	48/32	WFC + HET (four combination)	HET (four combination)	2	Clinical efficacy; GSS; TNF-α; IL-4; IL-33; EGF; VEGF; AE
Ye et al. (2024)	58.76 ± 8.35/64.20 ± 9.01	23/27	WFC	Rebamipide	12	Clinical efficacy; GSS; HPS; D-Dimer; PGI
Gao et al. (2022)	45.12 ± 0.83/48.24 ± 0.71	35/25	WFC + mosapride	Mosapride	16	Clinical efficacy; GSS; AE; TNF; IL
Zhang (2022)	51.53 ± 2.57/51.36 ± 2.63	90/66	WFC + PPI	PPI	8	Clinical efficacy; GSS; IL-8; TNF-α; SIL-2R; GAS; G-17; HPS
Hu (2021)	56.49 ± 5.71/56.54 ± 5.67	47/37	WFC + HET (four combination)	HET (four combination)	12	GAS; MTL; G-17; CD4; CD8; AE
Wang and Lv (2022)	N/A	80/72	WFC + PPI	PPI	12	Clinical efficacy; hemodynamic change; GSS; QOL
Liu (2022)	52.87 ± 4.66/52.43 ± 4.71	35/33	WFC + rebamipide	Rebamipide	4	Clinical efficacy; NO; CGRP; VEGF; PGE2; PCT; PGI; PGII
Wang (2021)	48.10 ± 7.92/47.23 ± 7.85	53/45	WFC + HET (four combination)	HET (four combination)	12	Clinical efficacy; GSS; IL-6; TNF-α; CRP; GAS; MTL; OPN; HSP-70; H. *pylori* eradiation rate
Liang et al. (2021)	44.14 ± 4.38/44.35 ± 4.46	35/25	WFC + HET (four combination)	HET (four combination)	2	Clinical efficacy; GSS
Du et al. (2021)	52.5 ± 5.6/52.3 ± 5.5	56/76	WFC + marzulene-S	WFC/marzulene-S	12	Clinical efficacy; HPS; EGF; EGFR positive rate
Bian et al. (2021)	53.90 ± 10.61/52.13 ± 11.53	31/29	WFC	Vitacoenzyme	24	HPS; clinical efficacy; GSS; gut microbiota abundance
Yao et al. (2020)	55.2 ± 7.2/55.6 ± 6.8	58/62	WFC + cinitapride	WFC	12	Clinical efficacy; GSS; GAS; MTL; GSH-Px; PGE2; AE
Li (2020)	48.03 ± 3.49/47.69 ± 3.81	47/23	WFC + mosapride	Mosapride	2	TNF-α; IL-1β; GAS; MTL; GSS
Fan (2020)	44.8 ± 12.5/<44.6 ± 12.7	58/47	WFC + PPI	PPI	8	Clinical efficacy; GSS
Wu (2020)	66.48 ± 10.83/65.52 ± 10.43	55/39	WFC + PPI	PPI	4	Clinical efficacy; SIL-2R; AE
Wang et al. (2020)	49.79 ± 2.63/49.82 ± 2.54	37/33	WFC + PPI	PPI	8	TNF-α; IL-8; IL-6; SOD; NO; AE
Long (2019)	46.24 ± 8.35/45.39 ± 8.24	51/45	WFC + HET (three combination)	Folic acid + HET (three combination)	24	Clinical efficacy; GAS; MTL
Song (2019)	48.62 ± 7.36/49.23 ± 6.98	53/33	WFC + PPI	PPI	4	Clinical efficacy; IL-1β; AE
Zhang et al. (2019)	56.75 ± 3.52/55.26 ± 3.99	52/36	WFC + H2RA	H2RA	16	Clinical efficacy; GSS; GAS; CGRP; VEGF; NO; AE
Zhu and Wei (2019)	45.6 ± 8.3/46.5 ± 9.4	108/76	WFC + probiotics	Probiotics	3	Clinical efficacy; GSS; AE
Lu et al. (2018)	41.8/42.8	119/81	WFC + HET (three combination)	HET (three combination)	4	Clinical efficacy; H. *pylori* eradiation rate; PGI; PGII
Xu et al. (2018)	44.32 ± 4.54/45.11 ± 4.76	151/89	WFC + HET (three combination)	HET (three combination)	8	Clinical efficacy; H. *pylori* eradiation rate; GSS; HPS
Liu and Zhang (2018)	46.7 ± 8.3/45.8 ± 7.9	79/85	WFC + mosapride	Mosapride	16	Clinical efficacy; GAS; MTL; TNF-α; IL-6; IL-8; GSS
Li and Zhao (2018)	41.8 ± 7.3/44.6 ± 4.9	51/41	WFC + probiotics	Probiotics	24	Clinical efficacy; GSS; H. *pylori* eradication rate; endoscopic assessment; HPS
Xiao et al. (2018)	61.38 ± 7.85/61.74 ± 8.15	44/42	WFC + PPI	PPI	24	Clinical efficacy; AE
Zhou et al. (2017)	47.01 ± 8.76/47.86 ± 9.43	49/37	WFC + folic acid	Folic acid	24	Clinical efficacy; GAS; MTL; HPS; AE
Liu et al. (2017)	51.22 ± 8.01/50.94 ± 9.21	58/55	WFC + folic acid	Folic acid	24	Clinical efficacy; Shh; Wnt3A; GSS; Shh/Wnt3A
He et al. (2017)	51.32 ± 5.41/50.64 ± 5.67	45/35	WFC + polaprezinc	Polaprezinc	12	Clinical efficacy; endoscopic assessment; TNF-α; IL-6; AE
Fan and Qiao (2017)	66.1 ± 10.3/65.4 ± 10.1	58/42	WFC + PPI	PPI	4	Clinical efficacy; TNF-α; IL-6; IL-8; AE
Yuan et al. (2016)	N/A	36/44	WFC	Placebo	12/24/36	Clinical efficacy; AE
Zhang et al. (2012)	42 ± 2.5	83/67	WFC + rebamipide	Rebamipide/WFC	12	Clinical efficacy; pathological efficacy; hemodynamic change
Lin (2011)	47.3 ± 12.9/45.1 ± 14.0	78/32	WFC	Vitacoenzyme	24	Clinical efficacy; hemodynamic change; ET; CGRP; AE
Fang and Du (2011)	48.4 ± 6.0/47.5 ± 6.2	49/41	WFC + teprenone	WFC + folic acid	12	Clinical efficacy; QOL; SF-36; AE
Nie and Sun (2011)	42.52 ± 5.76/42.52 ± 5.76	60/62	WFC + rebamipide	Rebamipide	12	Clinical efficacy
Lin et al. (2010)	47 ± 3	36/34	WFC + HET (three combination)	WFC	16	Clinical efficacy; recurrent rate; H. *pylori* eradiation rate; AE
Cao and Yang (2008)	42 ± 3	58/62	WFC + teprenone	WFC	12	Clinical efficacy; symptomatic efficacy; AE

Annotations.

### Risk of bias, heterogeneity, and publication bias

**FIGURE 2:**
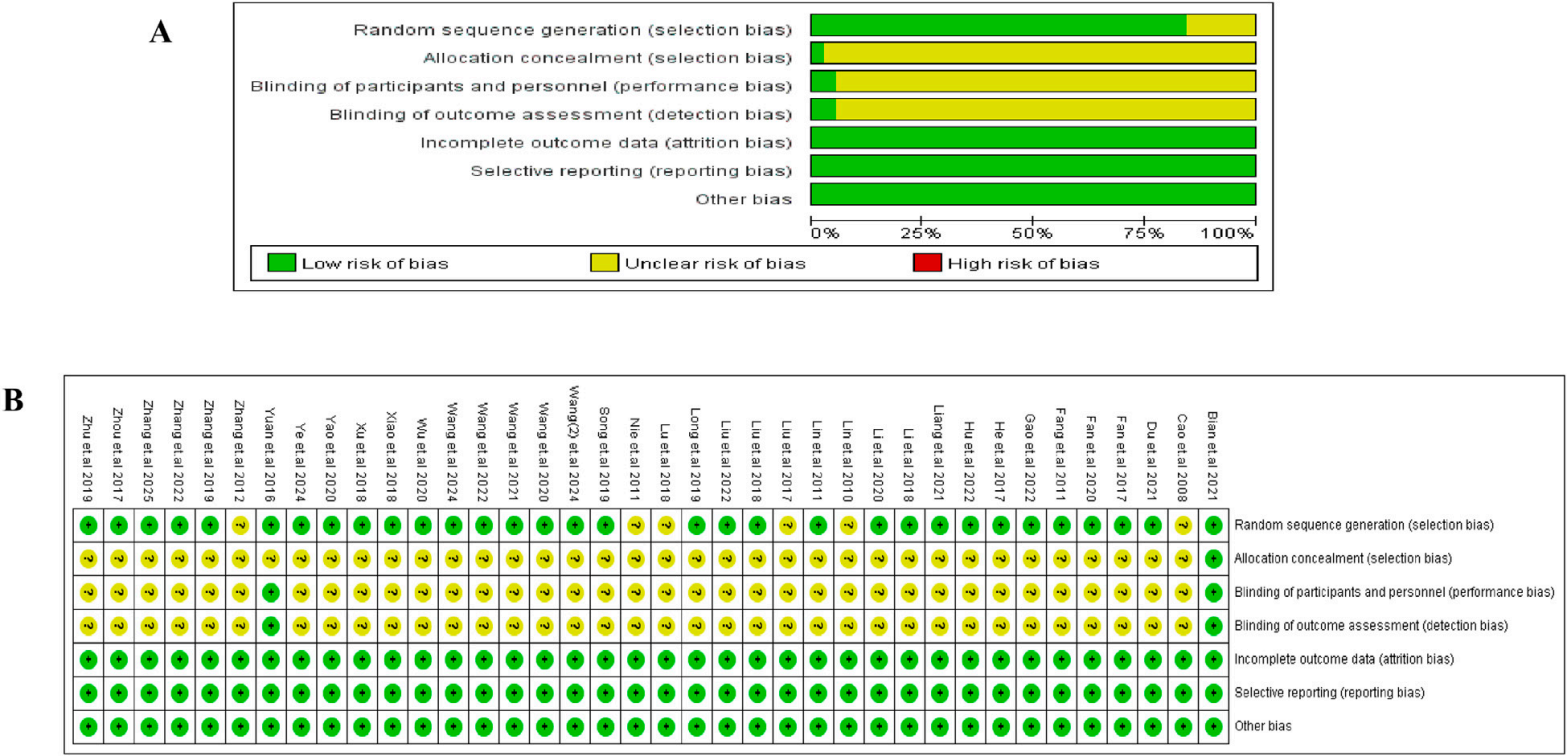
Risk-of-bias evaluation. **(A)** Risk of bias of each study. **(B)** Summary of the risk of bias.

The Cochrane Risk of Bias tool was used to evaluate the risk of bias in the included studies, and the detailed assessment is shown in Figure 2. Most trials reported adequate randomization (32/38, low risk), while only one described allocation concealment [15], and two mentioned blinding [15, 51]. Consequently, these domains were generally rated as “unclear risk.” All the studies were evaluated as having a “low” risk of bias in incomplete outcome data, selective reporting, and other bias domains.

**FIGURE 3:**
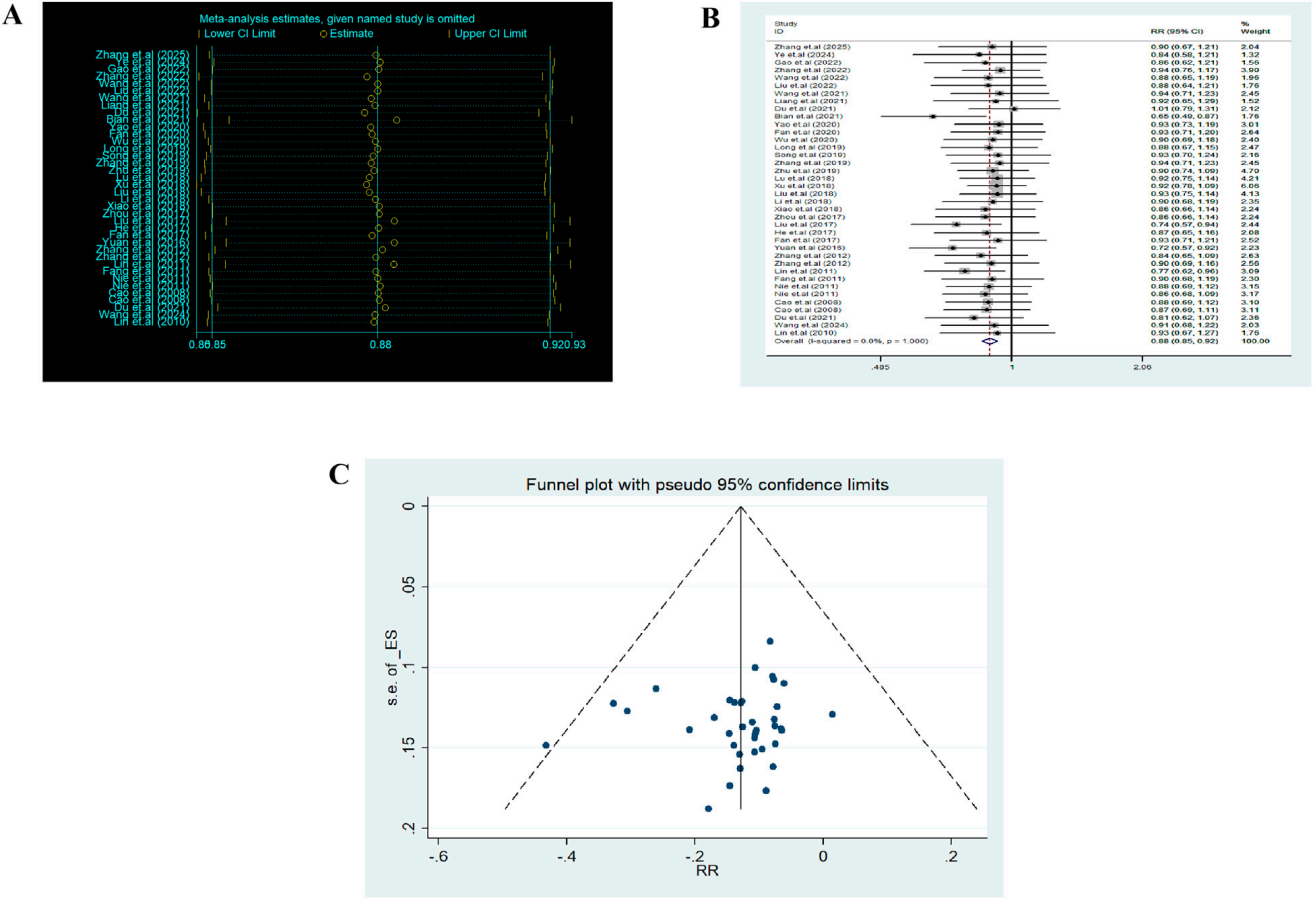
Heterogeneity analysis and funnel plot. **(A)** Sensitivity analysis. **(B)** Heterogeneity analysis. **(C)** Funnel plot.

Sensitivity analysis (Figure 3A) showed that removing any single study had a minimal impact on the pooled results (RR ≈ 0.88, 95% CI 0.86–0.92). Pairwise meta-analyses (Figure 3B) indicated negligible heterogeneity (I^2^ ≈ 0%). For the NMA, global inconsistency was assessed using the design-by-treatment interaction model, and local inconsistency was assessed using node-splitting analysis. No significant inconsistency was detected (*p* > 0.05), supporting the reliability of the network structure (Supplementary file 2).

The comparison-adjusted funnel plot appeared symmetrical, suggesting low publication bias (Figure 3C). Overall, the methodological quality of evidence was considered acceptable with low heterogeneity and strong robustness.

### Clinical efficacy

**FIGURE 4:**
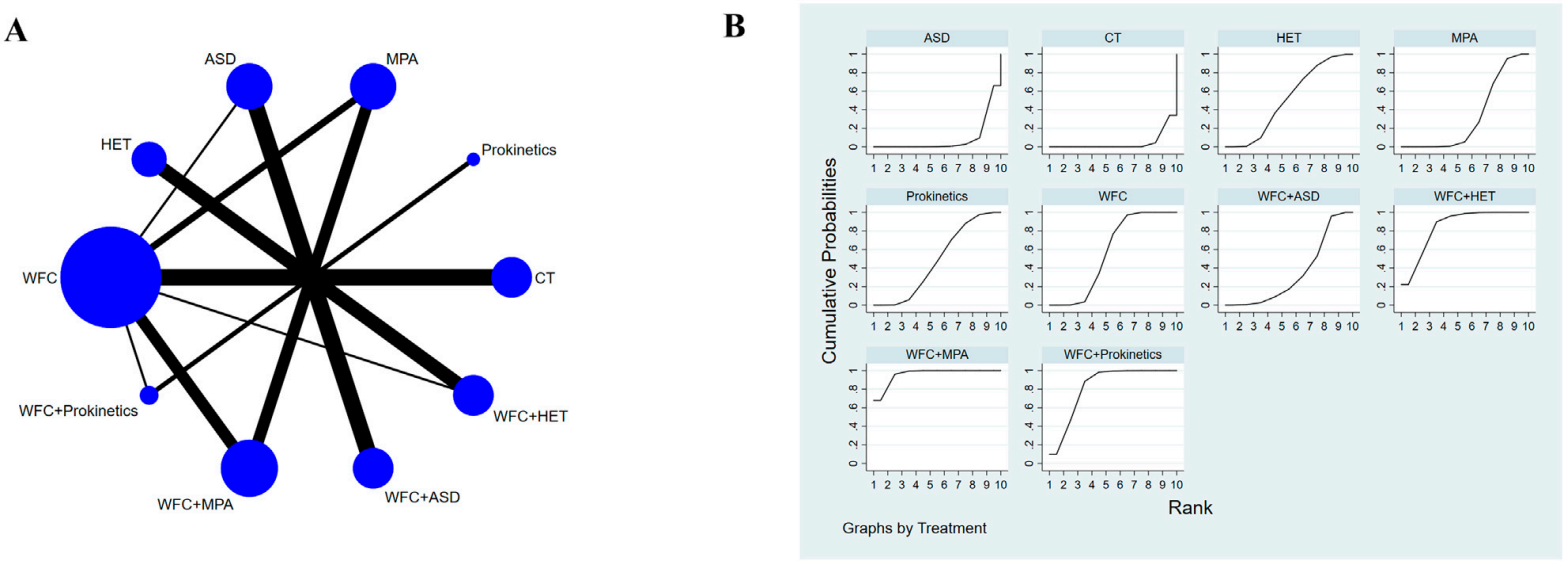
NMA results of clinical efficacy. **(A)** Network map. **(B)** SUCRA plot.

**FIGURE 5:**
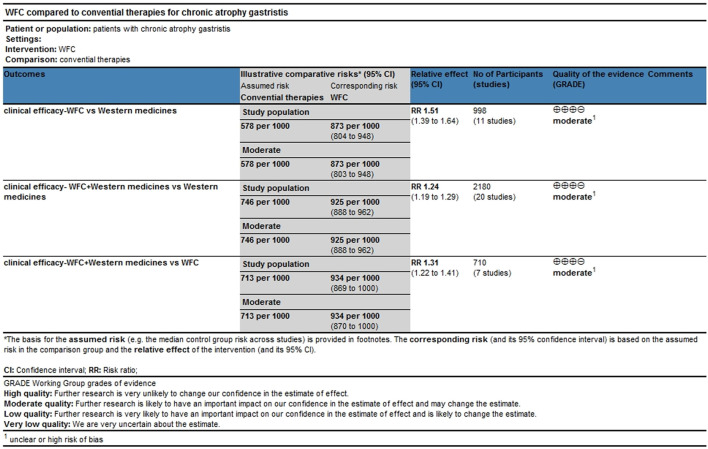
Grading of Recommendations Assessment, Development, and Evaluation framework.

A total of 34 studies involving 3,844 CAG patients were included in the assessment of clinical efficacy (Figure 4A). The results (Table 2) showed that WFC + MPA therapy was superior to WFC + ASDs, WFC alone, and any other single Western medicine (RR 1.29–1.80); WFC + prokinetics has a better efficacy than WFC + ASDs, WFC, and other Western medicine (RR 1.17–1.63); and WFC + HET was also superior to HET (RR = 1.20, 95% CI 1.13–1.28), MPAs (RR = 1.32, 95% CI 1.05–1.65), ASDs (RR = 1.58, 95% CI 1.17–2.13), and CT (RR = 1.66, 95% CI 1.32–2.07). These results suggested that WFC combined with Western medicine has a better therapeutic effect in treating CAG patients. In addition, using WFC alone was better than MPAs (RR = 1.11, 95% CI 1.01–1.22), ASDs (RR = 1.33, 95% CI 1.07–1.66), and CT (RR = 1.40, 95% CI 1.28–1.52). The SUCRA plot (Figure 4B) indicated that WFC + MPAs was the most effective therapy in inducing the clinical efficacy of CAG patients, followed by WFC + HET and WFC + prokinetics. The quality of evidence for this primary outcome was rated moderate (Figure 5). The main factors leading to downgrades included the risk of bias (inadequate blinding) and imprecision in small trials. No serious concerns were identified regarding inconsistency, indirectness, or publication bias.

**TABLE 2:** Risk ratio or the standard mean difference with 95% confidence interval of the reported outcomes.

Clinical efficacy									
WFC + MPA									
1.09 (0.87,1.35)	WFC + HET								
1.10 (0.94,1.30)	1.02 (0.79,1.31)	WFC + Prokinetics							
**1.29 (1.19,1.39)**	1.19 (0.96,1.46)	**1.17 (1.01,1.35)**	WFC						
**1.31 (1.04,1.65)**	**1.20 (1.13,1.28)**	1.18 (0.91,1.54)	1.02 (0.82,1.26)	HET					
**1.33 (1.10,1.61)**	1.23 (0.94,1.60)	**1.21 (1.10,1.33)**	1.03 (0.87,1.23)	1.02 (0.77,1.34)	Prokinetics				
**1.45 (1.15,1.84)**	1.34 (0.99,1.82)	**1.32 (1.01,1.72)**	1.13 (0.90,1.41)	1.11 (0.81,1.52)	1.09 (0.82,1.45)	WFC + ASD			
**1.43 (1.32,1.55)**	**1.32 (1.05,1.65)**	**1.30 (1.09,1.54)**	**1.11 (1.01,1.22)**	1.09 (0.86,1.39)	1.08 (0.88,1.31)	0.98 (0.77,1.26)	MPA		
**1.72 (1.36,2.16)**	**1.58 (1.17,2.13)**	**1.56 (1.20,2.02)**	**1.33 (1.07,1.66)**	1.31 (0.97,1.78)	1.29 (0.98,1.70)	**1.18 (1.11,1.25)**	1.20 (0.95,1.52)	ASD	
**1.80 (1.60,2.02)**	**1.66 (1.32,2.07)**	**1.63 (1.38,1.93)**	**1.40 (1.28,1.52)**	**1.38 (1.09,1.74)**	**1.35 (1.12,1.64)**	**1.24 (0.97,1.57)**	**1.26 (1.11,1.43)**	1.05 (0.83,1.32)	CT

Gastrin									
WFC + prokinetics									
1.10 (0.40,3.00)	WFC + HET								
**3.12 (1.01,9.66)**	**2.85 (1.71,4.74)**	HET							
**3.66 (2.21,6.07)**	**3.34 (1.08,10.31)**	1.17 (0.34,4.04)	Prokinetics						
**4.86 (2.41,9.81)**	**4.44 (2.15,9.16)**	1.56 (0.64,3.78)	1.33 (0.56,3.16)	WFC					
**14.42 (5.21,39.90)**	**13.15 (4.68,36.99)**	**4.62 (1.46,14.63)**	**3.94 (1.26,12.28)**	**2.96 (1.42,6.20)**	MPA				
**14.27 (5.20,39.16)**	**13.02 (4.67,36.31)**	**4.57 (1.45,14.37)**	**3.90 (1.26,12.06)**	**2.93 (1.42,6.07)**	0.99 (0.35,2.79)	CT			

Motilin									
WFC + prokinetics									
0.98 (0.28,3.42)	WFC + HET								
**2.64 (1.42,4.91)**	2.69 (0.67,10.84)	Prokinetics							
**3.03 (1.28,7.19)**	**3.09 (1.25,7.60)**	1.15 (0.40,3.32)	WFC						
3.12 (0.77,12.61)	**3.18 (1.70,5.96)**	1.18 (0.26,5.44)	1.03 (0.34,3.09)	HET					
**9.33 (2.67,32.63)**	**9.51 (2.65,34.11)**	3.53 (0.87,14.26)	**3.08 (1.24,7.62)**	2.99 (0.72,12.42)	MPA				
**9.51 (2.68,33.70)**	**9.69 (2.66,35.22)**	3.60 (0.88,14.71)	**3.14 (1.24,7.91)**	3.05 (0.73,12.81)	1.02 (0.28,3.72)	ASD			
**15.29 (4.35,53.81)**	**15.58 (4.32,56.23)**	**5.78 (1.42,23.50)**	**5.05 (2.02,12.60)**	**4.90 (1.17,20.47)**	1.64 (0.45,5.94)	1.61 (0.44,5.90)	CT		

Abdominal pain									
WFC + prokinetics									
1.46 (0.45,4.79)	WFC + HET								
2.74 (0.69,10.96)	1.87 (0.91,3.84)	HET							
**4.05 (2.52,6.52)**	2.77 (0.77,9.92)	1.48 (0.34,6.40)	Prokinetics						
**5.96 (2.69,13.17)**	**4.07 (1.69,9.80)**	2.17 (0.70,6.77)	1.47 (0.58,3.70)	WFC					
**7.12 (2.28,22.26)**	**4.86 (1.46,16.17)**	2.60 (0.64,10.53)	1.76 (0.51,6.04)	1.20 (0.53,2.71)	WFC + ASD				
**8.17 (3.13,21.32)**	**5.58 (1.99,15.65)**	2.98 (0.85,10.48)	2.02 (0.69,5.88)	1.37 (0.80,2.35)	1.15 (0.43,3.05)	CT			
**21.34 (6.78,67.12)**	**14.58 (4.36,48.73)**	**7.79 (1.91,31.72)**	**5.26 (1.52,18.20)**	**3.58 (1.57,8.19)**	3.00 (0.94,9.58)	2.61 (0.97,7.00)	MPA		
**27.07 (9.98,73.47)**	**18.49 (6.36,53.80)**	**9.88 (2.73,35.78)**	**6.68 (2.21,20.18)**	**4.55 (2.48,8.33)**	**3.80 (2.19,6.59)**	**3.31 (1.48,7.44)**	1.27 (0.46,3.53)	ASD	

Abdominal distension									
WFC + prokinetics									
1.73 (0.35,8.56)	WFC + HET								
**5.06 (1.56,16.47)**	2.93 (0.99,8.63)	WFC							
**8.86 (1.20,65.52)**	**5.12 (1.54,17.03)**	1.75 (0.35,8.81)	HET						
**13.19 (2.52,69.19)**	**7.63 (1.56,37.34)**	2.61 (0.81,8.34)	1.49 (0.20,10.91)	WFC + ASD					
**18.33 (4.43,75.75)**	**10.60 (2.78,40.39)**	**3.62 (1.64,7.97)**	2.07 (0.34,12.49)	1.39 (0.34,5.67)	CT				
**44.80 (10.44,192.30)**	**25.90 (6.53,102.75)**	**8.85 (3.76,20.80)**	5.06 (0.81,31.46)	**3.40 (1.54,7.48)**	2.44 (0.76,7.82)	ASD			

Acid reflux									
WFC + prokinetics									
1.62 (0.64,4.10)	WFC + HET								
**2.73 (1.38,5.40)**	1.68 (0.90,3.13)	WFC							
**4.70 (3.06,7.24)**	**2.90 (1.04,8.04)**	1.73 (0.77,3.87)	Prokinetics						
**11.51 (4.97,26.67)**	**7.08 (3.21,15.64)**	**4.22 (2.59,6.88)**	2.45 (0.95,6.28)	CT					
**11.53 (4.26,31.17)**	**7.09 (2.73,18.43)**	**4.23 (2.05,8.71)**	2.45 (0.83,7.24)	1.00 (0.42,2.40)	ASD				
**13.29 (4.78,36.96)**	**8.18 (3.06,21.87)**	**4.87 (2.28,10.43)**	2.82 (0.93,8.57)	1.15 (0.47,2.85)	1.15 (0.40,3.29)	MPA			

Adverse effect									
CT									
0.71 (0.12,4.28)	WFC								
0.72 (0.06,8.78)	1.01 (0.18,5.76)	WFC + ASD							
0.71 (0.03,19.17)	1.00 (0.06,15.81)	0.99 (0.04,25.70)	WFC Prokinetics						
0.55 (0.06,5.17)	0.77 (0.20,2.96)	0.76 (0.08,6.83)	0.77 (0.04,16.61)	HET					
0.41 (0.05,3.40)	0.57 (0.18,1.78)	0.56 (0.07,4.48)	0.57 (0.03,11.31)	0.74 (0.36,1.53)	WFC + HET				
0.36 (0.03,4.03)	0.50 (0.10,2.56)	**0.49 (0.27,0.89)**	0.50 (0.02,12.35)	0.65 (0.08,5.39)	0.88 (0.12,6.39)	ASD			
0.21 (0.01,7.08)	0.30 (0.01,6.05)	0.30 (0.01,9.50)	**0.30 (0.09,0.98)**	0.39 (0.01,10.49)	0.53 (0.02,13.04)	0.60 (0.02,18.32)	Prokinetics		
0.25 (0.02,2.44)	0.34 (0.08,1.45)	0.34 (0.04,3.23)	0.34 (0.02,7.73)	0.45 (0.06,3.20)	0.60 (0.10,3.76)	0.69 (0.08,6.05)	1.15 (0.04,32.06)	WFC + MPA	
0.18 (0.02,2.06)	0.25 (0.05,1.32)	0.25 (0.02,2.74)	0.25 (0.01,6.31)	0.33 (0.04,2.77)	0.44 (0.06,3.28)	0.51 (0.05,5.15)	0.85 (0.03,26.02)	0.74 (0.13,4.24)	MPA

### Gastrointestinal hormones

**FIGURE 6:**
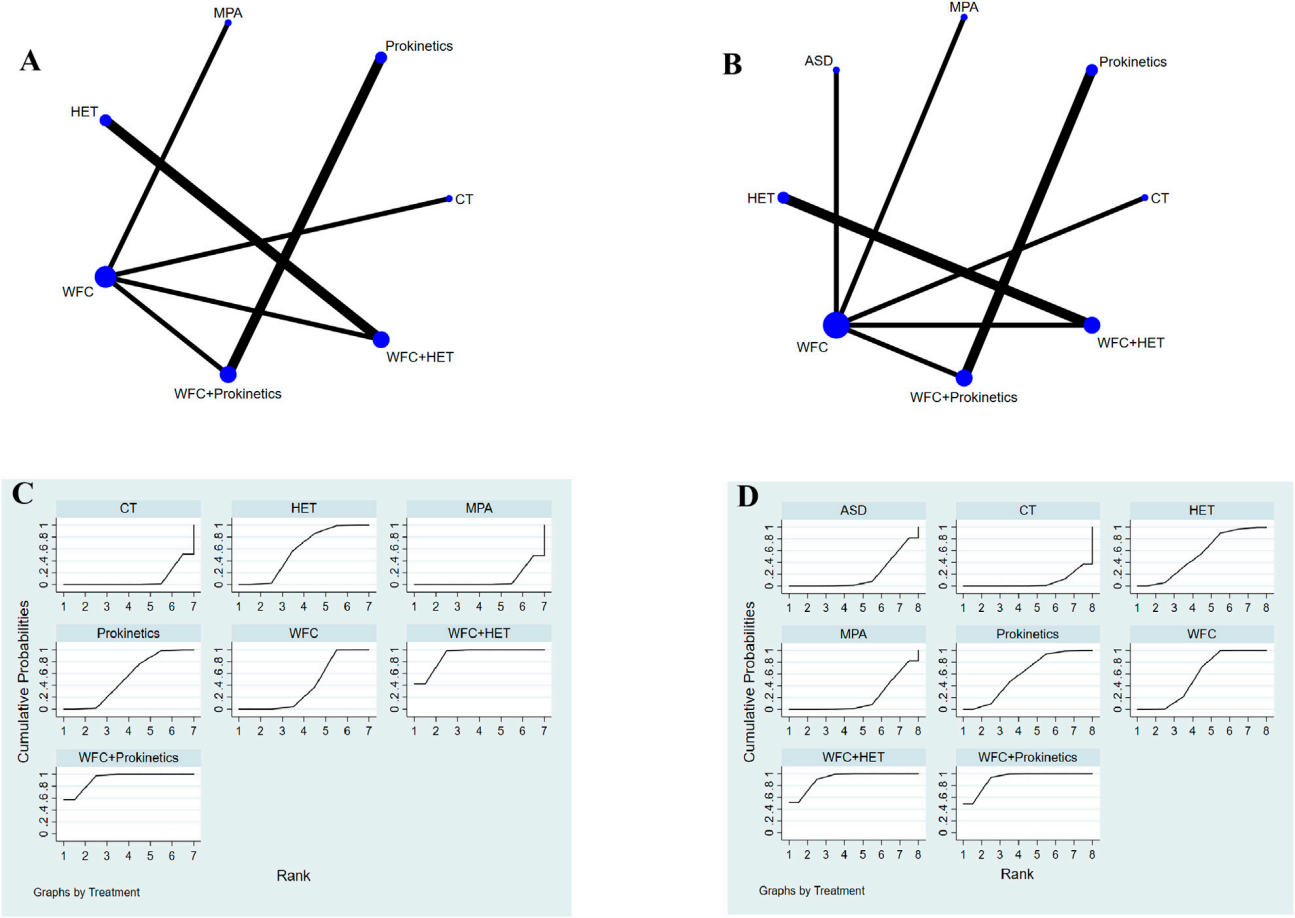
NMA results of gastrointestinal hormones. **(A)** Network map of gastrin. **(B)** Network map of motilin. **(C)** SUCRA of gastrin. **(D)** SUCRA of motilin.

A total of 11 studies involving 1,122 participants assessed changes in gastrointestinal hormones: GAS and MTL (Figures 6A,B). For the improvement of GAS (Table 2), WFC combined with prokinetics or HET was better than using HET, prokinetics, WFC, MPA, or CT alone (RR 2.93–14.42). In addition, HET, prokinetics, and WFC exhibited better performance than MPAs and CT (RR 2.93–4.57). For the improvement of MTL (Table 2), WFC + prokinetics was superior to prokinetics, WFC, MPAs, ASDs, and CT (RR 2.64–15.29), while WFC + HET was more effective than WFC, HET, MPAs, ASDs, and CT (RR 3.09–15.58). Moreover, using WFC alone also outperformed MPAs (RR = 3.08, 95% CI 1.24–7.62), ASDs (RR = 3.14, 95% CI 1.24–7.91), and CT (RR = 5.05, 95% CI 2.02–12.60). The SUCRA plots (Figures 6C,D) suggested that WFC combined with prokinetics was the best option for improving GAS and MTL levels in CAG patients, followed by WFC plus HET.

### Gastrointestinal symptoms

**FIGURE 7:**
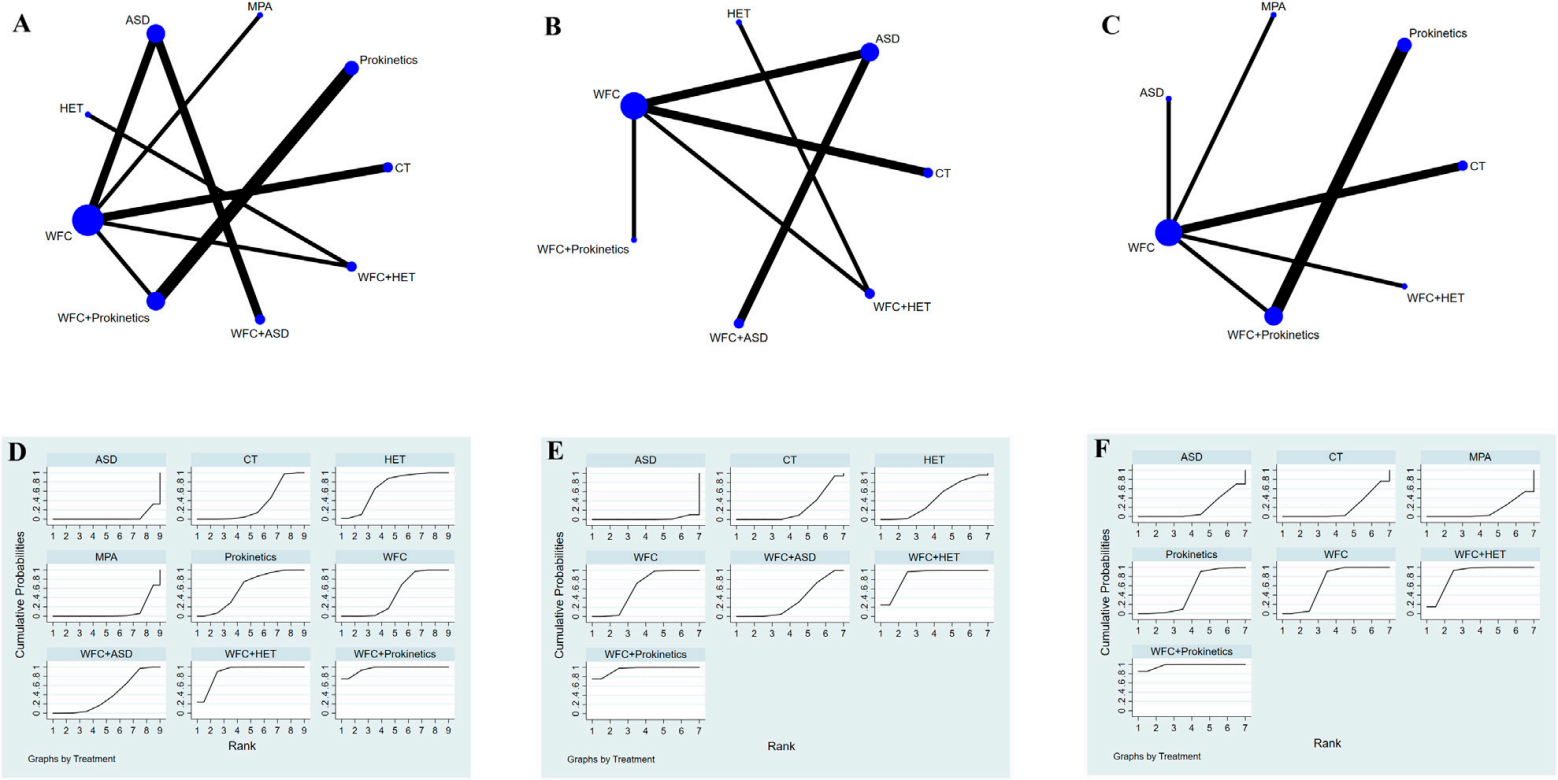
NMA results of gastrointestinal symptoms. **(A)** Network map of abdominal pain. **(B)** Network map of adbominal distension. **(C)** Network map of acid reflux. **(D)** SUCRA of abdominal pain. **(E)** SUCRA of abdominal distension. **(F)** SUCRA of acid reflux.

A total of 19 studies with 1,859 patients reported the improvement of gastrointestinal symptoms, 13 of which reported abdominal pain, 9 reported abdominal distention, and 9 reported acid reflux (Figures 7A–C). For relieving abdominal pain (Table 2), WFC combined with prokinetics was better than prokinetics, WFC, WFC + ASDs, CT, MPAs, and ASDs (RR 4.05–27.07), while WFC + HET was superior to WFC, WFC + ASDs, CT, MPAs, and ASDs (RR 4.07–18.49). In addition, HET, prokinetics, and WFC were all better than MPAs (RR = 7.79, 5.26, 3.58) and ASDs (RR = 9.88, 6.68, 4.55) in relieving abdominal pain. For alleviating abdominal distension (Table 2), WFC combined with prokinetics performed better than WFC, HET, WFC + ASDs, CT, and ASDs (RR 5.06–44.80), while WFC + HET was better than HET, WFC + ASDs, CT, and ASDs (RR 5.12–25.90). In addition, WFC showed a better effect than CT (RR = 3.62, 95% CI 1.64–7.97) and ASDs (RR = 8.85, 95% CI 3.76–20.80) in relieving abdominal bloating. For inhibiting acid reflux (Table 2), WFC combined with prokinetics was superior to prokinetics, CT, ASDs, and MPAs (RR 2.73–13.29) while using WFC alone had a better effect than CT (RR = 4.22, 95% CI 2.59–6.58), ASDs (RR = 4.23, 95% CI 2.05–8.71), and MPAs (RR = 4.87, 95% CI 2.28–10.43). The SUCRA plot (Figures 7D,E) suggested that WFC combined with prokinetics and WFC + HET ranked first and second in relieving abdominal pain and distension, along with inhibiting acid reflux, while WFC ranked third in alleviating abdominal bloating and acid reflux, and HET ranked third in reliving abdominal pain.

### Adverse effects

**FIGURE 8:**
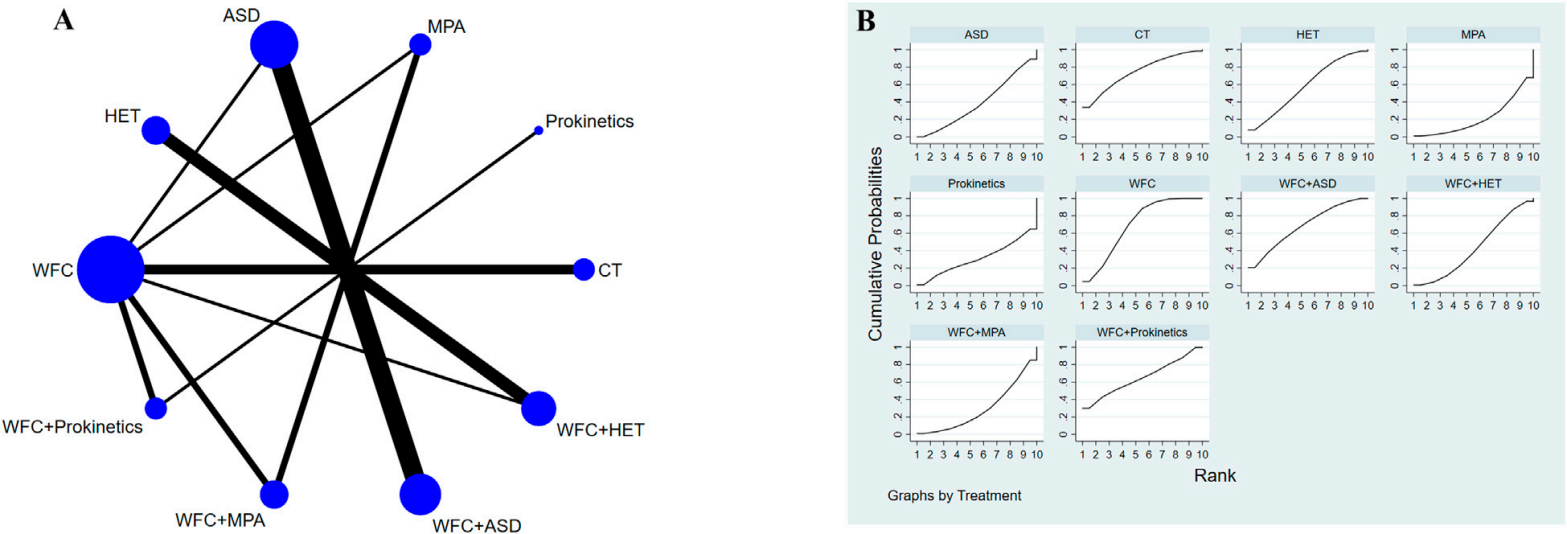
NMA results of adverse effects. **(A)** Network map. **(B)** SUCRA plot.

A total of 23 studies involving 2,175 participants reported adverse effects (Figure 8A). The results (Table 2) showed that WFC combined with ASDs or prokinetics was associated with a lower incidence of adverse effects than using ASDs (OR = 0.49, 95% CI 0.27–0.89) or prokinetics (OR = 0.30, 95% CI 0.09–0.98) alone. Other comparisons showed no significant safety differences, indicating that all regimens were similarly well-tolerated (Table 2; Figure 8B).

## Discussion

According to global data (Hahn et al., 2025), the pooled progression rates per 1,000 person-years for atrophic gastritis (AG), intestinal metaplasia (IM), and dysplasia were 2.09 (95% CI 1.46–2.99), 2.89 (2.03–4.11), and 10.09 (5.23–19.49), respectively. Notably, the study suggested that individuals with gastric precursor lesions—especially IM—face a similar risk of progression to gastric cancer (GC) regardless of geographic location, highlighting the importance of endoscopic surveillance and management of CAG. Currently, *H. pylori* eradication is used only as an adjunct for high-risk CAG patients, since eradication alone is insufficient to prevent progression (Kobayashi et al., 2015). Other treatments—such as PPIs, prokinetics, vitamin B12 supplementation, and mucosal-protective agents—also show limited efficacy and may cause adverse effects (Morgan et al., 2025). Therefore, exploring alternative and complementary strategies such as TCM formulations is of growing clinical interest.

This NMA demonstrates that WFC—a Chinese patent herbal formula consisting of *Panax ginseng* C.A.Mey., *Isodon amethystoides* (Benth.) H. Hara, and *Citrus aurantium* L.—exerts significant therapeutic benefits for CAG, particularly when combined with Western medical treatments. Combinations of WFC with mucosal-protective agents (WFC + MPAs), prokinetics (WFC + prokinetics), and *H. pylori* eradication therapy (WFC + HET) consistently outperformed monotherapies in improving clinical efficacy, regulating gastrointestinal hormones (gastrin and motilin), and relieving major symptoms such as abdominal pain, distension, and acid reflux. The SUCRA rankings indicated that WFC + MPAs was the most effective overall, while WFC + prokinetics showed the best improvement in gastrointestinal hormone levels and symptom relief. In addition, WFC-containing regimens were associated with fewer adverse events than the corresponding monotherapies, suggesting a possible protective role against drug-related toxicity.

Previous pairwise meta-analyses of WFC for CAG have generally focused on single comparisons (e.g., WFC versus conventional therapy or WFC + MPA versus MPA alone) and have yielded inconsistent results. Unlike these studies, our NMA provides an integrated comparative framework that incorporates both direct and indirect evidence across 10 interventions. This approach not only confirms earlier findings that WFC enhances therapeutic efficacy but also ranks the combination regimens, thereby providing clinicians with a hierarchy of optimal treatment strategies. Our findings align with earlier reports suggesting that WFC improves gastric mucosal function and immune modulation (Xie et al., 2024; Wang L. et al., 2023; Oyagi et al., 2010), but this study extends the evidence base by quantitatively demonstrating that WFC-based combinations outperform standard Western therapies.

The superior performance of WFC + MPAs may be attributed to the complementary mechanism. WFC exhibits anti-inflammatory and mucosal regenerative properties—suppressing the NF-κB pathway, reducing pro-inflammatory cytokines (IL-1β and TNF-α), and modulating immune cell responses through the regulation of TLR2 and CD14 expression (Xie et al., 2024; Wang B. et al., 2023). Its main component, *Panax ginseng* C.A.Mey., enhances local mucosal perfusion and reduces oxidative damage, while *Isodon amethystoides (Benth.)* H. Hara and *Citrus aurantium* L. promote glandular regeneration and modulate lipid and hormone metabolism (Oyagi et al., 2010; Dang et al., 2020; Wang et al., 2017). When combined with MPA, these effects likely synergize to accelerate mucosal healing.

Similarly, the observed superiority of WFC + prokinetics for improving gastrin and motilin levels may reflect WFC’s role in restoring gastrointestinal motility via MAPK pathway modulation and tryptophan metabolism (Wang et al., 2020; Wang et al., 2017). This mechanistic synergy could explain the marked relief of symptoms in abdominal bloating and reflux observed in our analysis. Notably, WFC monotherapy also showed significant efficacy compared to several single Western agents, suggesting intrinsic therapeutic potential through multiple pharmacodynamic pathways rather than symptom suppression alone. According to the product labeling and pharmacopoeia standards, WFC is contraindicated in pregnant women and individuals with known allergies to its components. Although no adverse drug interactions with Western medicines were reported in the included studies, comprehensive pharmacovigilance and pharmacokinetic investigations remain necessary.

However, several limitations should be noted. First, the methodological quality of the included studies varied, with many lacking detailed descriptions of allocation concealment and blinding, thus potentially introducing performance or detection bias. Second, moderate heterogeneity across studies—such as differences in patient characteristics, dosage regimens, and follow-up duration—may limit the generalizability of the findings. Third, indirect comparisons inherent to NMA depend on the transitivity assumption, and minor violations could affect the accuracy of the pooled estimates. Fourth, the predominance of small, single-center Chinese RCTs raises concerns about the potential overestimation of benefits. Although funnel plot analysis suggested low publication bias, it is important to acknowledge that most included studies were published in Chinese journals, where positive outcomes are more likely to be reported. Finally, most studies lacked long-term follow-up, precluding the assessment of sustained efficacy and safety.

Despite these limitations, the findings provide compelling evidence that integrating WFC with Western medicine—particularly with mucosal-protective agents or prokinetics—may optimize therapeutic outcomes for CAG. These regimens appear to not only enhance mucosal recovery and symptom control but also reduce adverse effects associated with prolonged pharmacotherapy. Future research should focus on large-scale, multicenter, and double-blinded RCTs that incorporate standardized diagnostic criteria, objective biomarkers of mucosal healing, and longer follow-up to confirm long-term benefits and mechanistic pathways.

## Conclusion

In conclusion, this network meta-analysis suggests that WFC, particularly when used in combination with conventional Western therapies, may provide additional clinical benefits for patients with CAG. The combination regimens appeared to improve the overall clinical efficacy, regulate gastrointestinal hormones, and reduce adverse events compared with monotherapies. Nevertheless, these findings should be interpreted cautiously as most included studies originated from Chinese settings and showed variable methodological quality. Further large-scale, multicenter randomized controlled trials with standardized protocols are needed to confirm these results, clarify WFC’s mechanisms of action, and evaluate its long-term safety and effectiveness in diverse populations.

## Data Availability

The raw data supporting the conclusions of this article will be made available by the authors, without undue reservation.

## References

[B1] Annibale B. Negrini R. Caruana P. Lahner E. Grossi C. Bordi C. (2001). Two-thirds of atrophic body gastritis patients have evidence of *Helicobacter pylori* infection. Helicobacter 6 (3), 225–233. 10.1046/j.1083-4389.2001.00032.x 11683925

[B2] Annibale B. Lahner E. Santucci A. Vaira D. Pasquali A. Severi C. (2007). CagA and VacA are immunoblot markers of past *Helicobacter pylori* infection in atrophic body gastritis. Helicobacter 12 (1), 23–30. 10.1111/j.1523-5378.2007.00467.x 17241297

[B3] Annibale B. Esposito G. Lahner E. (2020). A current clinical overview of atrophic gastritis. Expert Rev. Gastroenterol. Hepatol. 14 (2), 93–102. 10.1080/17474124.2020.1718491 31951768

[B4] Bian Y. Chen X. Cao H. Xie D. Zhu M. Yuan N. (2021). A correlational study of weifuchun and its clinical effect on intestinal flora in precancerous lesions of gastric cancer. Chin. Med. 16 (1), 120. 10.1186/s13020-021-00529-9 34801051 PMC8605594

[B5] Cao L. J. Yang X. F. (2008). Combined therapy with weifuchun and teprenone for chronic atrophic gastritis. J. Univ. South China Med Sci. 36 (05), 617–619.

[B6] Correa P. Piazuelo M. B. Wilson K. T. (2010). Pathology of gastric intestinal metaplasia: clinical implications. Am. J. Gastroenterol. 105 (3), 493–498. 10.1038/ajg.2009.728 20203636 PMC2895407

[B7] Dai Y. K. Zhang Y. Z. Li D. Y. Ye J. T. Zeng L. F. Wang Q. (2017). The efficacy of jianpi yiqi therapy for chronic atrophic gastritis: a systematic review and meta-analysis. PLoS One 12 (7), e0181906. 10.1371/journal.pone.0181906 28738092 PMC5524332

[B8] Dang Y. Liu T. Yan J. Reinhardt J. D. Yin C. Ye F. (2020). Gastric cancer proliferation and invasion is reduced by macrocalyxin C via activation of the miR-212-3p/Sox6 pathway. Cell. Signal 66, 109430. 10.1016/j.cellsig.2019.109430 31726103

[B9] Du Y. Bai Y. Xie P. Fang J. Wang X. Hou X. (2014). Chronic gastritis in China: a national multi-center survey. BMC Gastroenterol. 14, 21. 10.1186/1471-230X-14-21 24502423 PMC3922313

[B10] Du Q. Cai C. Y. Shi H. J. (2021). Effects of Marzulene-S combined with weifuchun on EGF and EGFR in HP-negative patients with chronic atrophic gastritis. Hainan Med. J. 32 (20), 2644–2647.

[B11] Eriksen M. B. Frandsen T. F. (2018). The impact of patient, intervention, comparison, outcome (PICO) as a search strategy tool on literature search quality: a systematic review. J. Med. Libr. Assoc. 106 (4), 420–431. 10.5195/jmla.2018.345 30271283 PMC6148624

[B13] Fan C. M. Qiao J. W. (2017). Curative efficacy of weifuchun tablets in combination with esomeprazol in treating elderly patients with chronic atrophic gastritis and its effects on serum inflammatory factors. China Med. Heral 14 (28), 125–128.

[B12] Fan Y. F. (2020). Clinical observation of weifuchun tablets combined with rabeprazole sodium enteric-coated capsules in treating chronic atrophic gastritis. China's Naturop. 28 (05), 65–66. 10.19621/j.cnki.11-3555/r.2020.0533

[B14] Fang H. Y. Du Y. C. (2011). Comparison of the efficacy of weifuchun combined with teprenone or folic acid in treating chronic atrophic gastritis. Zhejiang Pract. Med. 16 (03), 161–162. 10.16794/j.cnki.cn33-1207/r.2011.03.002

[B15] Gao J. Pan W. G. Yuan S. M. (2022). Efficacy analysis of weifuchun tablets combined with mosapride in treating chronic atrophic gastritis. Chin. Community Dr. 38 (17), 61–63.

[B16] Gu Z. Ling J. Cong J. Li D. (2020). A review of therapeutic effects and the pharmacological molecular mechanisms of Chinese medicine weifuchun in treating precancerous gastric conditions. Cancer Ther. 19, 1534735420953215. 10.1177/1534735420953215 32865036 PMC7466872

[B17] Guyatt G. H. Oxman A. D. Vist G. E. Kunz R. Falck-Ytter Y. Alonso-Coello P. (2008). GRADE: an emerging consensus on rating quality of evidence and strength of recommendations. BMJ 336 (7650), 924–926. 10.1136/bmj.39489.470347.AD 18436948 PMC2335261

[B18] Hahn A. I. Mulder D. T. Huang R. J. Zhou M. J. Blake B. Omofuma O. (2025). Global progression rates of precursor lesions for gastric cancer: a systematic review and meta-analysis. Clin. Gastroenterol. Hepatol. 23 (9), 1514–1524.e13. 10.1016/j.cgh.2024.09.003 39362617 PMC11958785

[B19] He D. L. Lin L. M. Lin C. X. (2017). Efficacy of weifuchun tablets combined with polaprezine granules in treatment of atrophic gastritis and effects on inflammatory cytokine level. Eval. Analysis Drug-Use Hosp. China 17 (05), 651–652. 10.14009/j.issn.1672-2124.2017.05.028

[B20] Hu J. Y. (2021). Effects of weifuchun combined with quadruple therapy on gastrointestinal hormones and immune function in patients with chronic atrophic gastritis. J Med Theor and Prac 34 (06), 956–958. 10.19381/j.issn.1001-7585.2021.06.023

[B21] Kobayashi M. Sato Y. Terai S. (2015). Endoscopic surveillance of gastric cancers after *Helicobacter pylori* eradication. World J. Gastroenterol. 21 (37), 10553–10562. 10.3748/wjg.v21.i37.10553 26457015 PMC4588077

[B23] Li S. H. Zhao Z. H. (2018). Clinical efficacy observation of weifuchun combined with bifidobacterium in treating patients with chronic atrophic gastritis. J. Bingtuan Med. 02, 25–28.

[B24] Li H. X. Liu Z. L. Han X. (2023). Expert consensus on the clinical application of weifuchun in treating precancerous lesions of chronic atrophic gastritis. J. Traditional Chin. Med. 64 (02), 212–216. 10.13288/j.11-2166/r.2023.02.019

[B22] Li Y. Z. (2020). Effects of weifuchun tablets combined with mosapride on chronic atrophic gastritis. Pract. J. Integr. Tradit. Chin. West Med. 20 (11), 71–72. 10.13638/j.issn.1671-4040.2020.11.034

[B25] Liang J. M. Luo X. M. Liao H. C. Lu D. (2021). Observation on the efficacy of quadruple therapy combined with weifuchun in the treatment of chronic atrophic gastritis. Strait Pharm. J. 33 (06), 140–141.

[B26] Lin H. (2011). Efficacy observation of weifuchun in treating precancerous lesions of chronic atrophic gastritis. Chin. J. Pharmacoepidemiol 20 (06), 286–288. 10.19960/j.cnki.issn1005-0698.2011.06.006

[B27] Lin Y. Cao D. Q. Qiu R. F. (2010). Efficacy observation of triple therapy for *Helicobacter pylori* eradication combined with weifuchun in treating chronic atrophic gastritis. J. Gannan Med. Univ. 30 (01), 48–49.

[B29] Liu R. Zhang L. H. (2018). Observation on efficacy of Weifuchun tablets combined with mosapride tablets in treatment of chronic atrophic gastritis. Eval. Analysis Drug-Use Hosp. China 18 (09), 1195–1197. 10.14009/j.issn.1672-2124.2018.09.012

[B30] Liu X. Y. Liu X. M. Yang Z. B. (2017). Clinical observation and mechanism of Weifuchun in treating gastric intestinal metaplasia. SH. J. TCM 51 (02), 44–47. 10.16305/j.1007-1334.2017.02.013

[B28] Liu Q. Q. (2022). Clinical efficacy of combined medication in treating Helicobacter pylori-associated chronic atrophic gastritis. Inn. Mong. Med. J. 54 (03), 328–329. 10.16096/J.cnki.nmgyxzz.2022.54.03.028

[B31] Long Y. (2019). Observation on the efficacy of Weifuchun tablets in treating chronic atrophic gastritis. J Med Theor and Prac 32 (07), 998–999. 10.19381/j.issn.1001-7585.2019.07.028

[B32] Lu D. W. Chen Y. Q. Liu S. L. Wang D. N. (2018). Clinical efficacy of weifuchun pill on Helicobacter pylori positive chronic atrophic gastritis and effects on pH and pepsinogen in gastric juice. World Chin. Med. 13 (09), 2182–2185.

[B33] Morgan D. R. Corral J. E. Li D. Montgomery E. A. Riquelme A. Kim J. J. (2025). ACG clinical guideline: diagnosis and management of gastric premalignant conditions. Am. J. Gastroenterol. 120 (4), 709–737. 10.14309/ajg.0000000000003350 40072510 PMC13166553

[B34] Nie M. Sun H. J. (2011). Observation on pathological improvement of chronic atrophic gastritis treated with weifuchun and rebamipide. J. Jilin Med. 32 (02), 265–266.

[B35] Notsu T. Adachi K. Mishiro T. Fujihara H. Toda T. Takaki S. (2019). Prevalence of autoimmune Gastritis in individuals undergoing medical checkups in Japan. Intern Med. 58 (13), 1817–1823. 10.2169/internalmedicine.2292-18 30918182 PMC6663548

[B36] Oyagi A. Ogawa K. Kakino M. Hara H. (2010). Protective effects of a gastrointestinal agent containing Korean red ginseng on gastric ulcer models in mice. BMC Complement. Altern. Med. 10, 45. 10.1186/1472-6882-10-45 20718962 PMC2936409

[B37] Page M. J. Mckenzie J. E. Bossuyt P. M. Boutron I. Hoffmann T. C. Mulrow C. D. (2021). The PRISMA 2020 statement: an updated guideline for reporting systematic reviews. BMJ 372, n71. 10.1136/bmj.n71 33782057 PMC8005924

[B38] Park J. Y. Cornish T. C. Lam-Himlin D. Shi C. Montgomery E. (2010). Gastric lesions in patients with autoimmune metaplastic atrophic gastritis (AMAG) in a tertiary care setting. Am. J. Surg. Pathol. 34 (11), 1591–1598. 10.1097/PAS.0b013e3181f623af 20975338

[B39] Pittman M. E. Voltaggio L. Bhaijee F. Robertson S. A. Montgomery E. A. (2015). Autoimmune metaplastic atrophic gastritis: recognizing precursor lesions for appropriate patient evaluation. Am. J. Surg. Pathol. 39 (12), 1611–1620. 10.1097/PAS.0000000000000481 26291507

[B40] Rugge M. Correa P. Dixon M. F. Fiocca R. Hattori T. Lechago J. (2002). Gastric mucosal atrophy: interobserver consistency using new criteria for classification and grading. Aliment. Pharmacol. Ther. 16 (7), 1249–1259. 10.1046/j.1365-2036.2002.01301.x 12144574

[B41] Savovic J. Weeks L. Sterne J. A. Turner L. Altman D. G. Moher D. (2014). Evaluation of the cochrane Collaboration's tool for assessing the risk of bias in randomized trials: focus groups, online survey, proposed recommendations and their implementation. Syst. Rev. 3, 37. 10.1186/2046-4053-3-37 24731537 PMC4022341

[B42] Shah S. C. Piazuelo M. B. Kuipers E. J. Li D. (2021). AGA clinical practice update on the diagnosis and management of atrophic gastritis: expert review. Gastroenterology 161 (4), 1325–1332.e7. 10.1053/j.gastro.2021.06.078 34454714 PMC8740554

[B44] Song J. H. Kim Y. S. Heo N. J. Lim J. H. Yang S. Y. Chung G. E. (2017). High salt intake is associated with atrophic gastritis with intestinal metaplasia. Cancer Epidemiol. Biomarkers Prev. 26 (7), 1133–1138. 10.1158/1055-9965.EPI-16-1024 28341758

[B43] Song Q. (2019). Clinical efficacy of weifuchun tablets combined with lansoprazole in treating chronic atrophic gastritis and its impact on serum IL-1β levels. Chin. Foreign Med. Res. 17 (17), 7–9. 10.14033/j.cnki.cfmr.2019.17.003

[B45] Sugano K. Tack J. Kuipers E. J. Graham D. Y. El-Omar E. M. Miura S. (2015). Kyoto global consensus report on *Helicobacter pylori* gastritis. Gut 64 (9), 1353–1367. 10.1136/gutjnl-2015-309252 26187502 PMC4552923

[B50] Wang B. Zhou W. Zhang H. Wang W. Zhang B. Li S. (2023). Exploring the effect of weifuchun capsule on the toll-like receptor pathway mediated HES6 and immune regulation against chronic atrophic gastritis. J. Ethnopharmacol. 303, 115930. 10.1016/j.jep.2022.115930 36403744

[B48] Wang D. S. (2024). Therapeutic effect of the combination of weifuchun tablets and lansoprazole in the treatment of chronic atrophic gastritis and its impact on the serum inflammatory index levels of patients. RARM 5 (05), 118–120.

[B49] Wang H. R. Lv S. L. (2022). Clinical observation of weifuchun capsules in treating atrophic gastritis with intestinal metaplasia. CJGMCM 37 (11), 1983–1986.

[B52] Wang H. Wu R. Xie D. Ding L. Lv X. Bian Y. (2020). A combined phytochemistry and network pharmacology approach to reveal the effective substances and mechanisms of wei-fu-chun tablet in the treatment of precancerous lesions of gastric cancer. Front. Pharmacol. 11, 558471. 10.3389/fphar.2020.558471 33381024 PMC7768900

[B47] Wang H. X. (2021). Observation on the efficacy of weifuchun tablets combined with rabeprazole quadruple therapy in patients with chronic atrophic gastritis and Hp infection. Acta Med. Sin. 34 (03), 22–26. 10.19296/j.cnki.1008-2409.2021-03-006

[B53] Wang H. Xie X. H. Bi Y. Z. Yu L. (2024). Clinical study on weifuchun combined with bismuth quadruple therapy for atrophic gastritis complicated with Hp infection. New Chin. Med. 56 (06), 85–89. 10.13457/j.cnki.jncm.2024.06.016

[B54] Wang L. Ding X. Li P. Zhang F. Ru S. Wang F. (2023). Efficacy and safety of weifuchun tablet for chronic atrophic gastritis: a systematic review and meta-analysis. PLoS One 18 (4), e0284411. 10.1371/journal.pone.0284411 37053262 PMC10101393

[B51] Wang S. Bao Y. R. Li T. J. Yu T. Chang X. Yang G. L. (2017). Mechanism of fructus aurantii flavonoids promoting gastrointestinal motility: from organic and inorganic endogenous substances combination point of view. Mag 13 (51), 372–377. 10.4103/pm.pm_179_16 28839359 PMC5551352

[B46] Wang W. G. (2020). Effects of weifuchun tablets combined with esomeprazole on IL-6, IL-8, and TNF-α levels in patients with chronic atrophic gastritis. World J. Complex Med. 6 (06), 178–180.

[B55] Wu Y. F. (2020). Clinical efficacy of weifuchun tablets combined with lansoprazole in treating chronic atrophic gastritis and its impact on serum soluble interleukin-2 receptor. Chin J Clin. Ration. Drug Use 13 (13), 55–56. 10.15887/j.cnki.13-1389/r.2020.13.028

[B56] Xiao X. Tan R. Y. Xiao D. (2018). Clinical efficacy and safety of weifuchun combined with esomeprazole in treating chronic atrophic gastritis. J. Med. Theor. Prac 31 (18), 2738–2739. 10.19381/j.issn.1001-7585.2018.18.019

[B57] Xie D. Wu C. Wang D. Nisma L. B. Liu N. Ye G. (2024). Wei-fu-chun tablet halted gastric intestinal metaplasia and dysplasia associated with inflammation by regulating the NF-κB pathway. J. Ethnopharmacol. 318 (Pt B), 117020. 10.1016/j.jep.2023.117020 37567428

[B58] Xu M. X. Peng B. Zhang C. J. Hou W. (2018). Clinical study on weifuchun in treating for chronic atrophic gastritis. Acta Chin. Med. 33 (08), 1537–1541. 10.16368/j.issn.1674-8999.2018.08.364

[B59] Yao P. Feng L. Hao L. L. Hu X. B. Wei J. J. (2020). Clinical study of cinitapride combined with weifuchun tablets in treating chronic atrophic gastritis. Drugs and Clin. 35 (12), 2441–2445.

[B60] Ye H. T. Liu J. X. Xu G. Liu D. B. Wang X. Y. Li L. (2024). Clinical observation of the efficacy of dahuang zhechong tablet combined with weifuchun capsule in the treatment of chronic atrophic gastritis with intestinal metaplasia. China Mod. Dr. 62 (07), 80–84.

[B61] Yuan L. L. Xin Y. Yan S. J. (2016). Study on the relationship between the course and the curative effect of the weifuchun capsule on the chronic atrophic gastritis. Clin. Med. 36 (11), 49–50.

[B63] Zhang J. W. Zeng S. P. Zhuang G. F. Li S. P. Zhang Q. P. (2012). Study on weifuchun and rebamipide combination therapy for chronic atrophic gastritis. Prog. Mod. Biomed. 12 (04), 696–698. 10.13241/j.cnki.pmb.2012.04.032

[B64] Zhang S. Zhu J. Chen Q. Zhang X. P. Wu X. B. (2019). Clinical study of weifuchun tablets combined with compound proglumide and cimetidine in treating chronic atrophic gastritis. Drugs and Clin. 34 (05), 1384–1388.

[B65] Zhang Y. Xu J. Yu H. L. (2025). Analysis of the curative effect of rebamipide combined with weifuchun in the treatment of patients with chronic atrophic gastritis. China Prac. Med. 20 (07), 75–78. 10.14163/j.cnki.11-5547/r.2025.07.019

[B62] Zhang S. X. (2022). Observation on the efficacy of weifuchun tablets combined with lansoprazole in treating patients with chronic atrophic gastritis. Mod. Med. Health Res. 6 (18), 81–84.

[B66] Zhou J. W. Ding S. L. Zhang W. X. (2017). Observation of folic acid tablets combined with weifuchun tablets in treating precancerous lesions of chronic atrophic gastritis. J. New Chin. Med. 49 (02), 42–44. 10.13457/j.cnki.jncm.2017.02.013

[B67] Zhu J. S. Wei Z. R. (2019). Clinical efficacy of bifidobacteria combined with weifuchun in treating chronic atrophic gastritis. Mod. Med. Health Res. 3 (01), 57–58.

