# Supplementary material for: Therapeutic effect and safety of Wei-Fu-Chun in the treatment of chronic atrophic gastritis: a network meta-analysis
Source: Front Pharmacol. 2025 Nov 20;16:1693427. doi: 10.3389/fphar.2025.1693427 (PMC12676455; doi:10.3389/fphar.2025.1693427)
Supplement: Supplementary file 1 [file Supplementaryfile1.docx]

Search strategy

("Chronic Atrophic Gastritis"[Mesh] OR "Atrophic Gastritis"[tiab] OR "Gastritis, Atrophic"[tiab] OR CAG [tiab])

AND

("Weifuchun"[tiab] OR "Wei Fu Chun"[tiab] OR "Wei-fu-chun"[tiab] OR "Stomach Reviving Tablet*"[tiab] OR "Stomach Reviving Capsule*"[tiab])

AND

("Proton Pump Inhibitors"[Mesh] OR "Histamine H2 Antagonists"[Mesh] OR "Anti-Bacterial Agents"[Mesh] OR "Cytoprotectants"[Mesh])

OR (PPI [tiab] OR omeprazole[tiab] OR lansoprazole[tiab] OR pantoprazole[tiab])

OR (H_2_RA [tiab] OR ranitidine[tiab] OR famotidine[tiab])

OR (sucralfate[tiab] OR rebamipide[tiab] OR polaprezinc[tiab])

OR ("bismuth quadruple therapy"[tiab] OR "amoxicillin"[tiab] OR "clarithromycin"[tiab])

AND

("Randomized Controlled Trial"[pt] OR "Controlled Clinical Trial"[pt] OR random*[tiab] OR placebo[tiab] OR "clinical trial"[tiab] OR RCT [tiab])
