# Supplementary material for: Therapeutic effect and safety of Wei-Fu-Chun in the treatment of chronic atrophic gastritis: a network meta-analysis
Source: Front Pharmacol. 2025 Nov 20;16:1693427. doi: 10.3389/fphar.2025.1693427 (PMC12676455; doi:10.3389/fphar.2025.1693427)
Supplement: Supplementary file 2 [file Supplementaryfile2.docx]

**Supplementary file 2. The node-spiliting analysis.**

| Side | Direct | | Indirect | | Difference | | | tau |
| --- | --- | --- | --- | --- | --- | --- | --- | --- |
|  | **Coef** | **Std.Err** | **Coef** | **Std.Err** | **Coef** | **Std.Err** | **P>\|z\|** |  |
| A F * | 0.335174 | 0.044036 | 0.009516 | 24.23261 | 0.325658 | 24.23265 | 0.989 | 1.94E-06 |
| B G * | 0.187242 | 0.048798 | 0.953151 | 163.7253 | -0.76591 | 163.7253 | 0.996 | 3.09E-07 |
| C F | 0.22521 | 0.071018 | 0.009537 | 0.063919 | 0.215673 | 0.095547 | 0.024 | 3.00E-09 |
| C H | 0.30702 | 0.046712 | 0.522694 | 0.08335 | -0.21567 | 0.095547 | 0.024 | 1.30E-08 |
| D F * | 0.287682 | 0.110378 | 0.429033 | 46.60398 | -0.14135 | 46.60411 | 0.998 | 2.03E-06 |
| D I * | 0.16607 | 0.030023 | -0.09941 | 104.1973 | 0.265482 | 104.1973 | 0.998 | 8.33E-08 |
| E J * | 0.185423 | 0.032941 | 1.024576 | 120.0901 | -0.83915 | 120.0901 | 0.994 | 5.60E-06 |
| F G * | 0.154151 | 0.073193 | -0.27906 | 80.52829 | 0.433208 | 80.52832 | 0.996 | 1.88E-08 |
| F H | 0.297484 | 0.043632 | 0.08181 | 0.085003 | 0.215674 | 0.095547 | 0.024 | 1.87E-08 |
| F J * | 0.169899 | 0.105566 | -0.26118 | 52.78468 | 0.431079 | 52.78479 | 0.993 | 9.89E-09 |

**Annotation:** A: Conventional therapies (CT); B: Prokinetics; C: Mucosal agents (MPA); D: Acid-suppressing drugs (ASD); E: H. pylori eradication therapy (HET); F: Wei-Fu-Chun (WFC); G: WFC+Prokinetics; H: WFC+MPA; I: WFC+ASD; J: WFC+HET.
